# Do potatoes and tomatoes have a single evolutionary history, and what proportion of the genome supports this history?

**DOI:** 10.1186/1471-2148-9-191

**Published:** 2009-08-07

**Authors:** Flor Rodriguez, Feinan Wu, Cécile Ané, Steve Tanksley, David M Spooner

**Affiliations:** 1 USDA, Agricultural Research Service; Department of Horticulture, 1575 Linden Drive, University of Wisconsin-Madison, Madison, Wisconsin 53706-1590, USA; 2 Department of Plant Breeding and Genetics, Department of Plant Biology, 248 Emerson Hall, Cornell University, Ithaca, New York 14853, USA; 3 Department of Statistics, Department of Botany, 1300 University Ave., University of Wisconsin-Madison, Madison, Wisconsin 53706-1590, USA

## Abstract

**Background:**

Phylogenies reconstructed with only one or a few independently inherited loci may be unresolved or incongruent due to taxon and gene sampling, horizontal gene transfer, or differential selection and lineage sorting at individual loci. In an effort to remedy this situation, we examined the utility of conserved orthologous set (COSII) nuclear loci to elucidate the phylogenetic relationships among 29 diploid *Solanum *species in the sister clades that include tomato and potato, and in *Datura inoxia *as a far outgroup. We screened 40 COSII markers with intron content over 60% that are mapped in different chromosomes; selected a subset of 19 by the presence of single band amplification of size mostly between 600 and 1200 bp; sequenced these 19 COSII markers, and performed phylogenetic analyses with individual and concatenated datasets. The present study attempts to provide a fully resolved phylogeny among the main clades in potato and tomato that can help to identify the appropriate markers for future studies using additional species.

**Results:**

Among potatoes, when total evidence is invoked, one single predominant history is highlighted with complete resolution within and among the three main clades. It also supports the hypothesis of the North and Central American B-genome origin of the tuber-bearing members of *Solanum *sect. *Petota *and shows a clear division between A genomes in clades 3 and 4, and B genomes in clade 1+2. On the other hand, when a prior agreement approach is invoked other potato evolutionary histories are revealed but with less support. These alternative histories could be explained by past hybridization, or fast rates of speciation. In the case of tomato, the analyses with all sequence data completely resolved 19 of 21 clades, for the first time revealed the monophyly of five clades, and gave further support for the recent segregation of new species from the former *Solanum peruvianum*. Concordance analyses revealed and summarized the extensive discordance among COSII markers. Some potential reasons for discordance could be methodological, to include systematic errors due to using a wrong model of sequence evolution, coupled with long branches, or mixtures of branch lengths within COSII, or undetected paralogy or alignment bias. Other reasons could be biological processes such as hybridization or lineage sorting.

**Conclusion:**

This study confirms and quantifies the utility of using DNA sequences from different parts of the genome in phylogenetic studies to avoid possible bias in the sampling. It shows that 11–18 loci are enough to get the dominant history in this group of *Solanum*, but more loci would be needed to discern the distribution of gene genealogies in more depth, and thus detect which mechanism most likely shaped the discordance.

## Background

The basic chromosome number in potatoes (sect. *Petota*), tomatoes (sect. *Lycopersicum*), and the most closely related outgroups (sects. *Etuberosum*, *Juglandifolium*, and *Lycopersicoides*) is *2n *= 2*x *= 24. Potatoes are alone in the group in possessing polyploids. Approximately 70% of the over 100 potato species are diploids, with most of the rest tetraploids (2*n *= 4*x *= 48) and hexaploids (2*n *= 6*x *= 72), with rare triploids and pentaploids [1].

Potatoes, tomatoes, and outgroups are characterized by relatively small chromosomes. Karyotypic and genomic analyses have included crossability success of interspecific combinations, hybrid sterility, hybrid viability, pollen fertility and in the degree of chromosomal homology [2-9]. Chromosome pairing relationships in interspecific hybrids and in polyploid potato species have been interpreted by genome formulae, although authors gave them different symbols. According to these hypotheses, five genomes (A, B, C, D and P) are recognized in the tuber-bearing species of section *Petota*. Genome symbol E was given to species of section *Etuberosum *based on the specificity of meiotic behavior and sterility of their diploid hybrids with the A-genome tuber-bearing potato species [7,10]. Symbol L was proposed for tomato (section *Lycopersicon*) on the bases of preferential chromosome pairing and clear-cut parental genome discrimination by using genomic *in situ *hybridization (GISH) and LLEE or artificial amphidiploids of tomato and *S. etuberosum *[11].

Wild and cultivated potatoes (*Solanum *L. sect. *Petota*) (see Additional file 1 for authors of taxa) grow from the southwestern United States to southern Chile. Hawkes [5] recognized 232 species in section *Petota*, and divided it into 19 tuber-bearing and two non-tuber-bearing series. He further divided these 19 tuber-bearing series into two superseries based on corolla morphology (superseries *Stellata *with stellate corollas and superseries *Rotata *with rotate corollas). He distinguished "primitive" and "advanced" forms of each superseries and recognized four groups based on morphological characters: primitive *Stellata*, advanced *Stellata*, primitive *Rotata*, and advanced *Rotata*. He hypothesized the evolution of the advanced *Rotata *morphology from primitive *Stellata*-like ancestors. He postulated that the ancestral wild potato species were diploid, possessed B genomes, produced white stellate corollas, and originated in North or Central America in the late Cretaceous to Eocene eras. Subsequent dispersal of one or more of these species to South America took place before the sinking of the Central American land bridge during the mid-Eocene to Pliocene eras, gradually leading to the evolution of species with A genomes and rotate corollas. He also postulated that when the bridge was restored in Pliocene times, a remigration of one or more of these diverged A genome species back into North and Central America allowed the hybridization and allopolyploidization with the native Mexican or Central American B genome taxa. This produced the tetraploid members of series *Longipedicellata *(AB). A second, comparatively recent migration of a second species (*S. verrucosum*, A genome) from South America formed the *Demissa *hexaploids (A_1_A_4 _[B,C,D]) [5] by crossing with series *Longipedicellata *tetraploids and possibly other series. In North and Central America are also another group of allopolyploids, members of series *Conicibaccata *(AC), but only series *Longipedicellata *was designated AB, and the source of the C and D genome donors of series *Conicibaccata *and *Demissa *is unknown. However, recent molecular clock data indicated that eggplant and tomato/potato shared a common ancestor approximately 14 MYA and potato and tomato 7 MYA [12]. So it seems unlikely that the timing for the events proposed by Hawkes really took place in the Cretaceous – Eocene eras but the events are still possible.

The latest taxonomic interpretation [13] recognized fewer species (190) and predicted even further reductions in species. Spooner et al. [14] used plastid DNA restriction site data and morphological data to reinvestigate the relationships of potatoes, tomatoes (former genus *Lycopersicon*), farther outgroups in *Solanum*, and other genera of the Solanaceae. Their results confirmed placement of all members of Hawkes's [5] tuber-bearing species into sect. *Petota *(90% bootstrap), but the non-tuber-bearing species were shown to be outgroups although with very low support (68%). Yet other plastid DNA restriction site marker data and DNA sequence data from plastids and single-copy nuclear DNA supported these relationships [15-18].

**Figure 1:**
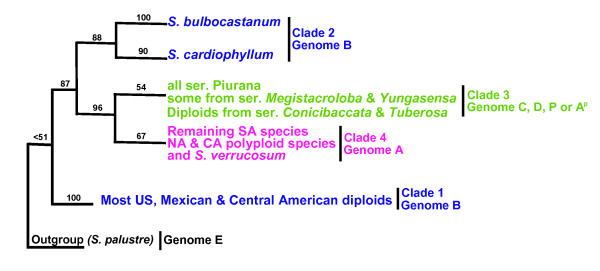
**Plastid clades of *Solanum *sect. *Petota ***[19-21,60].

Subsequent plastid DNA phylogenies documented section *Petota *(tuber-bearing) to be divided into four clades that often showed little relationship to Hawkes's taxonomic series (Figure 1) [19-21]. These clades contain: 1) North and Central American diploid species, with the exception of *S. bulbocastanum*, *S. cardiophyllum*, and *S. verrucosum*; 2) *S. bulbocastanum*, and *S. cardiophyllum*; 3) all examined members of series *Piurana *and some South American species belonging to other series (*S. andreanum, S. chancayense*, *S. immite*, *S. mochiquense *of ser. *Tuberosa*; *S. chomatophilum*, *S. colombianum*, *S. solisii, S. tundalomense *of ser. *Conicibaccata*; *S. huancabambense *of ser. *Yungasensia*; *S. sogarandinum *of ser. *Megistacroloba*); 4) all remaining South American species, *S. verrucosum *from Mexico, and North and Central American polyploid species. Only two clades were highly supported, clade 1 (100%) and clade 2 (88%), these clades contained all species with B genome, but they did not resolve together and the monophyly of the B genome was not supported. Clades 3 and 4 were poorly supported (54 and 67% bootstrap respectively), but their sister relationship was highly supported (96%). Single-copy granule-bound starch synthase (GBSSI or *waxy*) [22] and nuclear nitrate reductase gene data (*NIA*) [23] recovered the same four clades except united clades 1 and 2 as a single clade (72% in *waxy *and 92% in *NIA*), the nuclear phylogenies recognized the monophyly of B genome species (clade 1+2), and supported allopolyploid origins for some of the polyploids with species from different clades. The main difference between the two nuclear phylogenies was the relationship among main clades; in *NIA *clade 1+2 and 3 were sister in the most parsimonious tree although with very low support (≤51%), and *waxy *could not show any close relationships among the clades.

Taxonomic interpretations in tomato (*Solanum *sect. *Lycopersicon*) differed widely depending upon whether morphological or biological species concepts were used [24]. Rick et al. [25] recognized nine species of tomatoes, based mainly by their ability to intercross, and divided them into two crossing groups. Peralta et al. [24] summarized morphological studies of tomatoes [18,26-30], increased the traditional nine species of Rick et al. [25] to 13 species, and recognized tomatoes within *Solanum*. Tomato has two endemic species in the Galápagos Islands, and weedy escaped forms of *S. lycopersicum *are distributed worldwide. The remaining species and immediate tomato outgroup sections *Juglandifolia *(two species) and *Lycopersicoides *(two species) are endemic to western South America from Ecuador to northern Chile.

Peralta et al. [24] interpreted the many and sometimes conflicting morphological and molecular data sets to recognize four informal "species groups" within sect. *Lycopersicon*: the "Lycopersicon species group" including the four red to orange colored fruited species *S. cheesmaniae*, *S. galapagense*, *S. lycopersicum*, and *S. pimpinellifolium*; the "Neolycopersicon species group" containing only *S. pennellii*, the "Eriopersicon species group" containing *S. chilense*, *S. corneliomulleri*, *S. habrochaites*, *S. huaylasense*, *S. peruvianum *and, the "Arcanum species group" including *S. arcanum*, *S. chmielewskii*, and *S. neorickii*. They recognized sect. *Juglandifolia *as the sister group to sect. *Lycopersicon*.

In the past, comparative anatomy, cytogenetics, ecology and morphology had been used to infer phylogenetic relationships among taxa. Lately, with the development of molecular techniques, many fragments of the genome have been used in phylogenetic inference and multiple data sets have become available. As a result, phylogenetic studies now usually involve data sets from different gene regions, or different marker systems, or molecular and morphological data sets. There has been an active and controversial debate over the best ways to derive a phylogeny that incorporates all existing data. There are three different traditional approaches that have been used to analyze data from different sources: taxonomic congruence, character congruence and the prior agreement approach. They all seek to maximize evidence, the first one from the relationships (cladograms) using the summaries of data as evidence, and the other two derived directly from characters using data observation as evidence [31].

Proponents of the consensus approach argue that partitions should not be combined before estimating the phylogenetic tree; rather, the trees should be estimated separately from each partition and combined using taxonomic congruence [32]. The decision to keep data sets separate generally reflects a hypothesis that either 1) different evolutionary processes are acting on different data sets, or 2) different data sets reflect different evolutionary histories [33].

Character congruence, also known as the total evidence approach, involves all the data available for a group of terminal taxa. The goal is to seek a single, best-fitting hypothesis, which in cladistics involves maximizing character congruence [31]. The prior agreement approach or "conditional combination" tests for character incongruence before analysis, and only combines the data when no significant character incongruence exists between partitions.

Farris et al. [34] proposed the Incongruence Length Difference (ILD) test that many researchers use to assess whether or not data sets should be combined. The idea is to determine if there is more incongruence between data sets than is expected between similarly sized random partitions of a homogeneous data set.

Baum [35] proposed that the identification of the primary concordance tree (PCT) could be a valuable summary of the dominant phylogenetic history among a group of organisms. The PCT is composed of clades with higher concordance factors (CF, proportion of the genome for which a given clade is true) than any contradictory clade. Ané et al. [36] developed a Bayesian-based methodology that estimates the distribution of evolutionary histories within a multi-gene data set and summarizes the results allowing genealogical information from one gene to influence our estimates of another gene's genealogy. It also allows the identification of genes with outlier gene-tree topologies that permits estimation of the proportion of the genome that was transferred during a possible introgression event. Baum [35] pointed out the possible outcomes that can arise if reticulation events had happened during the evolutionary history of a group of organisms. If a single event of horizontal gene transfer (HGT) had occurred, one primary history and one minor history are expected. Under lineage sorting or introgression, one primary history and co-minor histories are expected. But, if hybridization occurs two co-primary histories are expected. Thus, the concordance approach is considered a modified consensus tree method that allows the identification of different phylogenetic histories in the face of reticulation, where estimated CFs are metrics that give an indirect measure of reticulation. Furthermore, it can be assumed that high CFs provide evidence that relationships among the organisms under study have been mostly divergent, and that low CFs are indicative of reticulate evolution. Baum [35] stressed that the acquisition of a divergence structure increases gradually as a result of gene lineage extinction in reproductively isolated populations or demes. Thus, the speciation process reaches its ultimate point when a CF of 1 is reached. Finally, he stated that a clade which has a CF that is greater than all contradictory clades has some genealogical unity that indicates a history of genetic isolation and suggested that this clade can be formally named.

Recently, single- to low-copy nuclear DNA markers have been explored for phylogenetic reconstruction. Here we test a specific subset of these markers called conserved ortholog set II (COSII) markers in the sister clades potato and tomato [37]. Orthologs are genes sharing a common ancestor by descent, in contrast to paralogs that are duplicated copies within a genome through polyploidization or tandem duplications [38,39].

The purpose of our study is to test a diverse range of COSII markers and data analyses for phylogenetic reconstruction in the sister clades tomato and potato. The phylogenetic relationships between and among species from these two groups have been the subject of study by many researchers, but these relationships remain unresolved because of the use of a limited number of molecular markers. Our study was greatly aided by COSII conversion into a set of consensus PCR primers that amplify these orthologues in the Solanaceae, referred to as universal primers for euasterid I (UPA) [37]. UPAs are designed to amplify either intronic regions (iUPA primers) that provide the polymorphism needed for the proposed tomato and potato study, or exonic regions (eUPA primers) that are more appropriate for comparisons of more distantly related species. Our study uses many more molecular markers than prior phylogenetic studies in the tomato and potato clades. It does not attempt to provide a definitive study of the evolutionary relationships within these clades, but rather to identify appropriate markers for future studies that should incorporate a wider species sampling. Our approach consists of examining 1) diverse markers on all 12 linkage groups, 2) amplification of a single band (in diploids) of representative taxa, 3) markers with length between 600–1200 bp that amplify with single pass sequencing, and 4) markers with over 60% intron content appropriate for this taxonomic level of closely related species.

## Results

### Allelic variation and sequence alignment

Of the 30 species analyzed, four tomato species had two alleles: *S. chilense *and *S. huaylasense *in COS5c and COS13b, *S. peruvianum *and *S. habrochaites *in COS7b, *S. huaylasense *in COS10b, *S. peruvianum *in COSX2 and COS11b, *S. chilense *in COS1b; *S. chilense*, *S. peruvianum *and *S. habrochaites *in COS8b, and *S. chilense*, *S. peruvianum *and *S. huaylasense *in COS15b (16 cases in total). In four of these 16 cases alleles from the same species form a clade; in four cases alleles formed polytomies; and in eight cases alleles from the same species formed a clade with another species [40].

Complete sequences (total missing data less than 0.3%) were generated for the 19 COSII. In general, the farthest outgroups, *Datura inoxia *and *S. dulcamara*, showed the longest sequence and the tomato species and their closest outgroups showed more gaps in aligned sequences in comparison with potato [40].

### Model selection

In general, likelihood models that account for rate variation, allowing for gamma-distributed rate variation among sites (Γ), resulted in the greatest increase in Maximum Likelihood (ML). In more than 90% of the cases the HKY model that includes five parameters best fitted the data [40]. In cases where the two criteria for selection chose different models, the model that is implemented in Mr. Bayes was preferred.

### Phylogenetic analysis of individual loci

Analysis of individual COSII sometimes showed incongruent results. To illustrate this we discuss our data using maximum parsimony analysis in potato and direct the reader to [40] for details in the tomato and complete data sets and with maximum likelihood and Bayesian inference. In the potato data set the number of parsimony informative sites per individual COSII varied from 13 to 64; in tomato, from 20 to 286; and for the potato and tomato data set from 46 to 317. All data sets showed high consistency, retention and rescaled consistency indices, with consistency indices ranging from 0.753 to 1.000, and rescaled consistency indices ranging from 0.612 to 1.000. There was a moderate level of homoplasy (calculated by H = 1-CI) with less than 25% for all loci.

In the potato data set seven of the 12 COSII sequenced supported the three main potato clades as a monophyletic group. In eight of the 12 COSII clade 1+2 (*S. bulbocastanum*, *S. polyadenium*, *S. stenophyllidium*, *S. trifidum*) was monophyletic with bootstrap support over 54%; in three of the 12 COSII, clade 1+2 failed to resolve into a distinct clade; one COSII placed *S. polyadenium *outside of clade 1+2. When we forced the monophyly of clade 1+2 for this COSII, the shortest tree was ten steps longer than the unconstrained tree, which was significant by the Templeton Test (*p *= 0.0016–0.0075) and the monophyly of this clade was rejected [40].

Clade 3 (*S. albornozii*, *S. andreanum*) had bootstrap support more than 50% in nine of the 12 COSII; one COSII failed to group these species in their own clade, and two COSII grouped one of the two species in clade 4. Forcing these species to be monophyletic resulted in trees only three and two steps longer than the unconstrained tree, and the Templeton test failed to reject their monophyly (*p *= 0.3657 and *p *= 1). Thus, stochastic noise alone can explain the misplacement of these species.

Clade 4 (*S. brevicaule*, *S. raphanifolium*, *S. verrucosum*) had bootstrap support more than 59% in ten of the 12 COSII; in the remaining two COSII *S. raphanifolium *did not form a clade with the other two species, and trees forcing all three species to group together were only one and three steps longer than the Maximum Parsimony (MP) unconstrained tree. The Templeton test failed to reject their monophyly and revealed that the entire clade 4 became unresolved in trees that were one and three steps longer than the MP tree. This indicates that the observed non-monophyly is not significant (*p *= 0.8635 and *p *= 0.0833–0.3173 respectively) [40].

Regarding the relationships among the three main clades, four of the 12 COSII supported clade 1+2 as sister to the rest of potatoes, with bootstrap support ranging from 59–100%. Three COSII placed clade 3 as sister to the rest of potatoes and only two of these three COSII gave bootstrap support of 69% and 90%. Two COSII placed clade 4 as sister to the rest of potatoes with a bootstrap support of 60% and 100% respectively. The remaining three COSII did not support any of the three basal relationship alternatives in their most parsimonious trees. Attempts to force species to follow either of these three possibilities were not rejected for COS2c, because constrained trees were only four or five steps longer than the MP unconstrained tree. In contrast, COS5 could not reject clade 1+2 as sister to the other potatoes, and COS5c could not reject clade 3 species as sister to the others.

### Combined data analyses

We explored three approaches to combine the data: total evidence, prior agreement, and the consensus approach.

#### Total evidence, potato data set

**Figure 2:**
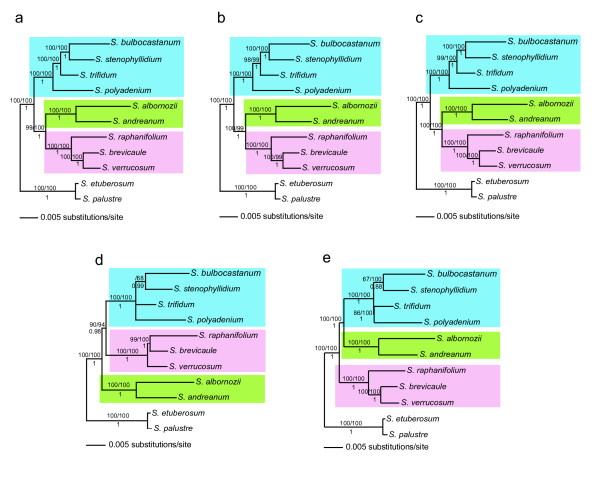
**Potato Bayesian phylogram based on a combined analysis**. Using 12 COSII sequences in (a), six COSII (COS 9, COS 9b, COS 11, COS 3, COS 8 and COS 3c) sequences (b), three COSII (COS 9, COS 9b and COS 11) sequences (c), with COS7b (d), and with two COSII (COS10b and COS1c) (e). Branch lengths are drawn in proportion to the estimated number of substitutions per site and represent an average of the branch length of all trees sampled in the Markov chain that have that branch. Bootstrap values higher than 50% are indicated above branches, the first value refers to Maximum Parsimony and the second to Maximum Likelihood analyses; below branches are the posterior probability values. Species belonging to clades 1+2 are in blue shadow, species in clade 3 in green shadow and species in clade 4 with pink shadow.

We concatenated the 12 COSII of the potato data set into a data matrix of 10,886 characters, with introns accounting for 85% of the total concatenated length. The concatenated alignment contained 1,219 (11.2%) variable sites, of which 519 (4.8%) were parsimony informative. Phylogenetic analyses of the concatenated sequence using MP, ML and BI, all yielded a single fully resolved tree with high bootstrap support and Bayesian posterior probability (Figure 2a).

#### Total evidence, tomato data set

**Figure 3:**
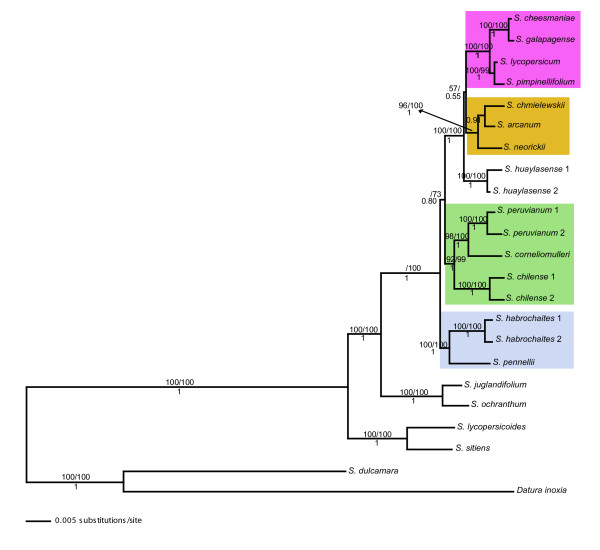
**Tomato Bayesian phylogram based on a combined analysis of 18 COSII sequences**. *Datura inoxia *and *S. dulcamara *were used as outgroups. Numbers after the species name indicate allelic variants. Support values are placed as in Figure 1. Clades with many species have a background for helping follow the results and discussion.

Eighteen COSII were concatenated; the data matrix contained 15,709 characters, 82% of these representing introns, with the total data set containing 4,022 variable sites of which 1,910 (12.2%) were parsimony informative. MP, ML and BI supported at least the same 18 of 21 nodes as completely resolved with very high bootstrap values (MP: 90–100%, ML: 99–100%) and posterior probability (*PP *= 1) (Figure 3). Two nodes were not very well supported in MP, ML and BI, one of them was the placement of the clade formed by *S. arcanum*-*S. chmielewskii*-*S. neorickii *(51% in MP, < 51 in ML, and 0.55 in BI). In MP and BI this clade was resolved with the red-orange tomatoes (*S. cheesmaniae*, *S. galapagense*, *S. lycopersicoides*, *S. pimpinellifolium*), and in ML it resolved as sister to a clade containing the red-orange tomatoes and *S. huaylasense*. The second unresolved node was the placement of the clade formed by *S. chilense*-*S. corneliomulleri*-*S. peruvianum*; ML and BI revealed that this clade was closer to the red-orange tomatoes than the clade formed by *S. habrochaites*-*S. pennellii *(73% in ML and 0.80 in BI). On the other hand, MP showed that *S. habrochaites*-*S. pennellii *was closer to the red-orange clade than the clade *S. chilense*-*S. corneliomulleri*-*S. peruvianum *(55%). Finally, MP and ML resolved *S. chmielewskii *and *S. neorickii *with very low support (< 51%). On the other hand, BI resolved *S. arcanum *and *S. chmielewskii *with a posterior probability of 0.91; the terminal branch that resolved *S. arcanum*-*S. chmielewskii*-*S. neorickii *had 96%, 99% (bootstrap) and 1 (posterior probability) support in MP, ML and BI respectively.

#### Total evidence, potato and tomato data set

**Figure 4:**
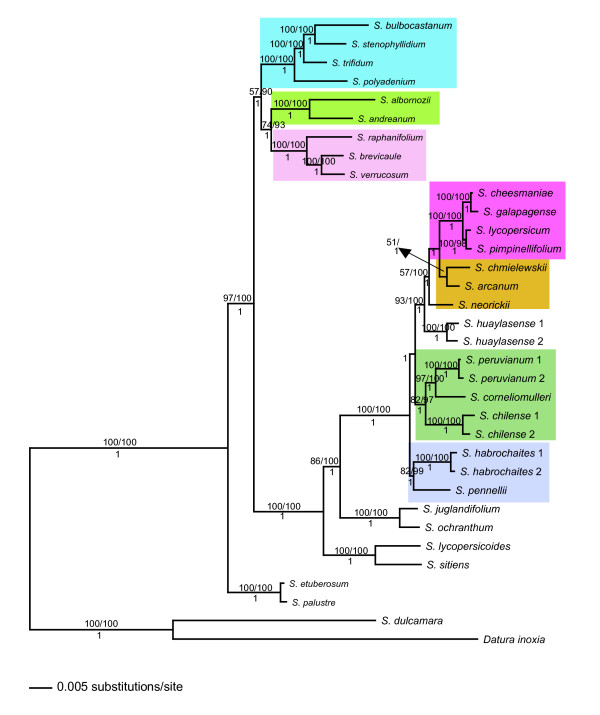
**Bayesian phylogram based on a combined analysis in potato, tomato and outgroups**. *Datura inoxia *and *S. dulcamara *were used as outgroups. Numbers after the species name indicate allelic variants. Support values are placed as in Figure 1. Shading as in Figures 2, 3.

After the concatenation of 11 COSII, the matrix contained 11,566 characters; 3,006 were variable and 1,799 (15.6%) were parsimony informative. Bayesian inference showed a completely resolved phylogeny. On the other hand, both MP and ML had two nodes with very low support (≤51%): 1) the placement of the clade with *S. arcanum*, *S. chmielewskii*, and *S. neorickii*, and 2) the node that showed relationships among these three species. Additionally, in MP the potato clade had only 57% bootstrap support (Figure 4).

#### Prior agreement, potato data set

A prior agreement approach combines only congruent data and excludes data partitions with a significant level of incongruence, as they can introduce error that can obscure reliable data and lead to erroneous topologies [41,42]. We explored the congruence of the different gene phylogenies in potato using ILD tests. From the 12 original COSII in potatoes, 66 pairwise comparisons were made with a level of significance of 0.00076 (= 0.05/66), and nine of them showed a high level of discordance (*p *= 0.0006). Three of these COSII (COS2c, COS5c and COS7b) counted for eight of these nine discordances (*p *= 0.0004), and the last one was between COS11 and COS10b (*p *= 0.0006).

Through reiterative analyses we proceeded to eliminate discordant COSII and constructed a set of six COSII that were highly congruent and produced highly resolved topologies similar to the entire concatenated data set and named this "6COScon" (COS 9, COS 9b, COS 11, COS 3, COS 8 and COS 3c) Rodriguez [40]. MP, ML, and BI of 6COScon gave the same phylogeny as the 12 COSII combined with 100% of bootstrap in all nodes except one that was supported at 99% (Figure 2b).

We further explored a minimum number of COSII to be used in a large number of species without losing resolution by further reducing the "6COScon" to three COSII by choosing the best three by two criteria: 1) length of the sequence, and 2) number of parsimony informative sites and called this "3COSa" (COS9, COS9b, COS11). MP, ML, and Bayesian analyses produced phylogenetic results almost identical to the 12 COSII set (Figure 2c).

#### Consensus approach, potato data set

**Figure 5:**
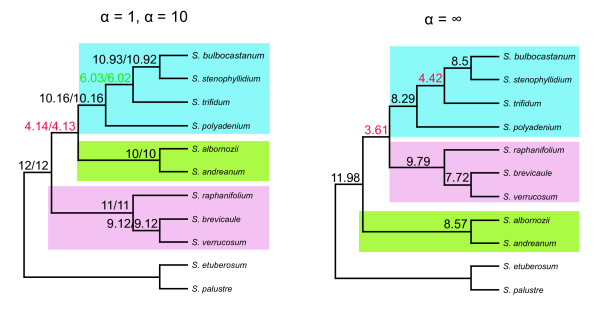
**Results from potato Bayesian concordance analysis using 12 COSII**. Number of COSII supporting each clade indicated above branches (concordance factors), in red are clades supported by less than 50% of the COSII; in green clades supported by almost 50% of COSII and in black clades supported by more than 50% COSII. Shading as in Figure 2.

**Figure 6:**
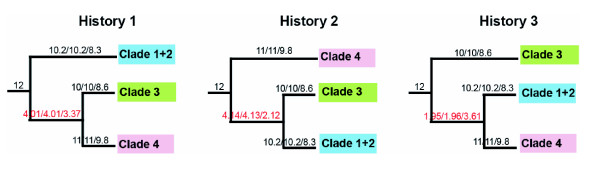
**Summary of the Bayesian concordance analysis in potatoes performed with 12 COSII**. Above branches are the concordance factors for α = 1, 10 and infinite respectively. The numbers supporting the branches in red are clades supported by less than 50% of the COSII and in black clades supported by more than 50% COSII. Shading as in Figure 2.

Figure 5 shows the potato primary concordance trees and Figure 6 shows the concordance factors of all three resolutions for the placement of the main potato clades. With all three choices of prior (α = 1, 10 and infinite) there was over 95% confidence that clade 1+2 (CF 10.16 = 85% of COSII), clade 4 (CF 11 = 92%) and clade 3 (CF 10 = 83%) formed clades for the majority of COSII. However, each of the three resolutions for the placement of those clades received concordance factors below 50% with over 95% credibility: the discordance among COSII markers was inferred to be real and strong. With all priors, the credibility intervals for the CF of all three resolutions (Figure 6) overlapped, therefore, it is still uncertain which placement is favored by the largest proportion of COSII.

#### Consensus approach, tomato data set

**Figure 7:**
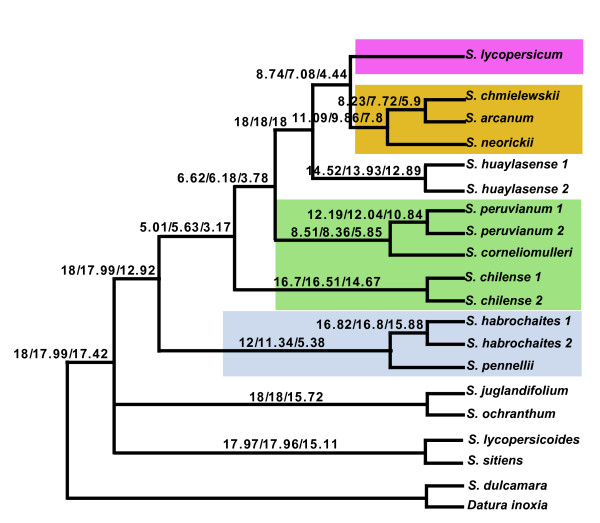
**Summary of the Bayesian concordance analysis in tomatoes with all 18 COSII**. Above branches are the concordance factors for α = 1, 10 and infinite respectively. Shading as in Figure 3.

In the first concordance analysis performed with the tomato data set, the tomato clade was supported as monophyletic for almost all COSII in all runs and with all α. Results from α = 1 and 10 placed sect. *Lycopersicoides *as the closest tomato outgroup to the tomato clade with just 50% of COSII supporting this relationship (9.31 and 9.22 COSII respectively); the other 50% of the COSII supported sect. *Juglandifolia *as the closest tomato outgroup. On the other hand, for α infinite, which corresponds to assuming that all gene trees are independent, and no concordance is assumed a priori, sect. *Juglandifolia *was placed as the closest tomato outgroup for only 40% of the COSII (7.21 COSII); sect. *Lycopersicoides *was supported as the closest outgroup to tomato for 32.5% of COSII (5.85 COSII). Section *Juglandifolia *and *Lycopersicoides *resolved together in only 9.3% of COSII, and the remaining 18% of COSII supported other relationships that could be considered as noise. Even though sect. *Lycopersicoides *was supported by a little more than 50% of the COSII as the closest outgroup to tomato with α = 1 and 10; and sect. *Juglandifolia *was supported by at least 40% of the COSII and by more COSII than any other contradictory clade with α infinite, the confidence intervals for all these CF overlapped. We conclude that sect. *Juglandifolia *and sect. *Lycopersicoides *are the closest tomato outgroups but concordance analysis cannot distinguish which is sister to the tomato clade for most of the COSII markers. Given that we have two primary histories that have the support of 50% of the genes with α = 1 and 10, hybridization could be invoked as the main driving force [35] but since more than two histories are revealed with α infinite more testing of the concordance approach should be done and more genes should be sampled before we can conclude with confidence that hybridization or introgression was the main driving force (Figure 7).

Results from the second tomato concordance analysis placed the clade consisting of *S. habrochaites *and *S. pennellii *as sister to the rest of tomatoes, indicating that those are the most divergent group; the CF was 30% (5.38 COSII) with α infinite, 63% (11.34 COSII) for α = 10 and 66% (12 COSII) for α = 1, and the credibility intervals for the CF of conflicting clades did not overlap. Thus, we conclude that *S. habrochaites *and *S. pennellii *are the most divergent tomatoes and are monophyletic (Figure 7). This analysis also placed *S. corneliomulleri *with *S. peruvianum *in all the runs with CF of 33% to 47% of COSII, and *S. chilense *outside of the clade conformed by *S. corneliomulleri*-*S. lycopersicum*-*S. peruvianum*, with CF ranging from 18% to 32% (Figure 7).

In the last concordance analysis with the tomato data set, all 18 COSII supported *S. huaylasense *as the sister to two clades, one formed by the red-orange colored fruit tomatoes (*S. cheesmaniae*, *S. galapagense*, *S. lycopersicoides*, *S. pimpinellifolium*) and the other formed by three species, *S. arcanum*-*S. chmielewskii*-*S. neorickii*; it also resolved *S. chmielewskii *with *S. arcanum *in all three α values (Figure 7).

## Discussion

As was pointed out in the background, all three approaches summarizing multiple data sets seek to maximize evidence, but there are questions about when each approach should be applied. Combined analysis of multiple data sets is justified when each data set has evolved under the same underlying history, in which differences in the estimated tree are due only to sampling error or model mis-specification [35]. Under this assumption, the combined phylogenetic analysis improves the signal to noise ratio. It potentially allows for more accurate estimation of the single shared genealogy. It also produces a more accurate phylogeny than from a consensus approach. But, in cases where genes have tracked more than one underlying history, some of the differences among data sets would not be due to sampling error, but to genealogical discordance. In this case we may wish to estimate the primary history with information quantifying the extent to which different genes have followed that history. That primary history can be obtained using a combined analysis if there are similar amounts of phylogenetic signal in each sampled gene. But, because combined analysis assumes that there is only one evolutionary history, the discordance is assumed to be caused by homoplasy, and when reticulation has occurred the inferred tree may not resemble any of the underlying histories. In such a case, Bayesian concordance analysis (BCA) would be a better procedure to use, because it does not assume one single evolutionary history. Rather, it allows us to make statements of statistical confidence in the estimated concordance factors by taking into account the prior evidence of discordance, uncertainty in gene tree estimates, and the number of genes sampled. It also allows the investigation of potentially interesting biological processes, such as incomplete lineage sorting, hybridization, and lateral gene transfer [36]. For this reason, BCA is becoming a tool to analyze genealogies that are neither fully reticulate nor fully divergent [35]. Therefore, we followed all three approaches to get insights into the evolutionary processes in tomato and potato.

COSII analyses support many aspects of prior phylogenetic interpretations of potato and tomato, show some new relationships, but most notably, when total evidence is invoked, one single predominant history is highlighted with complete resolution in potato (Figure 2a) and nearly so in tomato (Figure 3). In potato, the four clades discovered from plastid DNA restriction site data [19,21] is supported except, like *waxy *sequence data [22] and *NIA *sequence data [23] clades 1 and 2 are united into a single clade. Results from total evidence highly support the hypothesis of a North and Central American B-genome origin of the tuber-bearing members of *Solanum *section *Petota*, as it was shown to be sister of the rest of potatoes. It resolves the controversy among all previous individual gene sequencing studies that showed different relationships among main clades. On the other hand, when a prior agreement or consensus approach was invoked, other evolutionary histories in potatoes are revealed but with less support (Fig 2d, 2e, Figures 6, 7). Of interest is that the branches showing these two alternative histories (Figure 2d, 2e) are shorter than the branch showing the predominant evolutionary history (Figure 2a). These alternative histories could be explained by past hybridization events or fast rates of speciation.

In tomato, 19 of the 21 nodes are completely resolved and for the first time this study highly supports the monophyly of five clades: 1) a clade that includes *S. arcanum*-*S. chmielewskii*-*S. neorickii*, 2) a clade conformed by *S. chilense*-*S. corneliomulleri*-*S. peruvianum*, and the sister relationship between *S. corneliomulleri *and *S. peruvianum*; 3) a clade formed by *S. habrochaites*-*S. pennellii *as basal in tomatoes, 4) a clade that includes *S. cheesmaniae *and *S. galapagense*, and 5) a clade formed by *S. lycopersicum *and *S. pimpinellifolium *(Figure 3). COSII data also clearly support the segregation of *S. peruvianum *sensu lato into at least three species, *S. arcanum*, *S. huaylasense*, *S. peruvianum*+*S. corneliomulleri *(on the same clade) as a possible single species, or all four of these species, following Peralta et al. [43]. The data show *S. arcanum *and *S. huaylasense *to be very distinct from *S. corneliomulleri *and *S. peruvianum *that were on the same clade (Figure 3). The two branches that remain unresolved are the placement of the clade *S. arcanum*-*S. chmielewskii*-*S. neorickii *and the relationship among these three species. Both of these branches are very short (Figure 3), indicating the possible need for additional markers as was pointed out by Zou et al. [44], who showed the need for 142 gene sequences to resolve two small branches in the phylogeny of rice. The sister group relationships of sect. *Lycopersicon *to sect. *Juglandifolia *and to sect. *Lycopersicoides *were completely resolved thereby clarifying ambiguities in previous studies.

Concordance analyses in tomatoes reveal the same relationships as total evidence, but the monophyly of the clade with *S. chilense*-*S. corneliomulleri*-*S. peruvianum *is not supported and the placement of sect. *Juglandifolia *or sect. *Lycopersicoides *as the closest tomato outgroup remained unresolved. Furthermore, concordance analyses reveal and summarize the extensive discordance among COSII markers. This discordance is precisely why a large sample of nuclear loci was necessary for the reconstruction of a robust phylogeny in potato and tomato. Some potential reasons for discordance could be methodological, to include systematic errors due to using a wrong model of sequence evolution, coupled with long branches, or mixtures of branch lengths within COSII, or undetected paralogy or alignment bias. Other reasons could be biological processes such as hybridization or lineage sorting or fast speciation.

In the complete data set, where potatoes and tomatoes are analyzed together, the sister relationship between the potato and tomato clades is highly supported in all analyses and sect. *Etuberosum *is supported as sister to both (100%, 100% and 1), confirming these controversial relationships as was first shown by Spooner et al. [14]. Additionally, our study highlights the importance of high intron content (more than 60% in this study), and PCR amplification length between 600 and 1000 bp to be useful to investigate relationships among potato and potato. It shows the importance of intensive screening of COSII primers to maximize their utility in potato and tomato and allows us to propose the use of a reduced set of COSII primers for maximum efficiency. For example, for the major clades of sect. *Petota*, three COSII (COS9, COS9b and COS11) were enough to reveal the predominant history with high support, and we suggest beginning with these COSII in future studies in potato. This suggestion has to be taken with caution because when more taxa and more accessions per taxon are sampled the resolution can be reduced. Finally, the utility of gaps as additional characters in phylogenetic studies is not questionable but it is advisable to use them when their inclusion does not increase homoplasy. However, in this study, gap characters usually increased phylogenetic signal.

## Conclusion

COSII sequence data are useful for potato and tomato phylogenetic studies. Intron contents more than 60% were the best to investigate relationships within this group of closely related species. When total evidence is invoked, a well-supported predominant history is revealed in both potato and tomato. In the case of potato, with the prior agreement and consensus approaches, two additional possible histories were revealed, although with less statistical support. We show that 11–18 loci are enough to get the dominant history in potato and tomato respectively, but a reduced set of three COSII provide the same phylogeny in potato. Finally, we determined that there is not a single evolutionary history for potato and tomato, as at least three different possible histories were revealed. More loci would be needed to discern the distribution of gene genealogies in more depth, and thus detect which mechanism most likely shaped discordance among individual COSII.

## Methods

### Plant Materials and DNA Isolation

One diploid genotype from nine wild potato species (sect. *Petota*) representing all four plastid clades, all 13 wild tomato species (sect. *Lycopersicon*), both species of sect. *Juglandifolia*, both species of sect. *Lycopersicoides*, two species of sect. *Etuberosum*, and two farthest outgroups, *Datura inoxia *and *S. dulcamara *were analyzed (30 species in total; see Additional file 1). These species were chosen to cover most of the genomic groups and clades in potato, all of the species recognized in tomato [24], and appropriate outgroups. Leaf total DNA from each species was isolated using a standard CTAB protocol [45]. DNA quality and quantity were estimated by comparison with CsCl-purified λ DNA digested with *Pst*I on ethidium bromide-stained agarose gels.

### Choice, amplification, and sequencing of COSII

We tested 40 COSII loci that were putatively single copy in tomato and potato, from all 12 tomato and potato chromosomes (see Additional file 2). We screened these 40 COSII in genomic DNA of four tomato and four potato species. PCR amplifications were performed using 20 μL reactions consisting of 0.1 μM final concentration of each primer (see Additional file 2) and 20 ng of template genomic DNA. Amplifications were carried out in an MJ Research DNA Engine Dyad^® ^Peltier Thermal Cycler (Watertown, Massachusetts) using an initial denaturation at 94°C for 5 min, followed by 35 cycles of 94°C for 30 s, 55°C for 1 min, 72°C for 2 min and with a final elongation at 72°C for 10 min. The reactions were run on a 1.5% agarose gel with 1× TBE buffer for 3 hours. The above PCR conditions worked for most COSII but in some cases we had to modify these PCR conditions (see Additional file 3).

We selected 19 of these 40 loci based on single band amplification and length of the PCR product mostly between 600–1200 bp and with more than 60% intron content (see Additional file 3), and amplified DNA in all 30 species. To clean the product for sequencing the reaction was digested with EXO-SAP-IT^® ^(USB Corp., Cleveland OH) following the manufacturer's instructions except that reaction volumes were halved. One μL of this product was sequenced with the same primers in a 5 μL reaction using the ABI Big Dye dideoxynucelotide termination kit (Applied Biosystem, Foster City, California). Amplifications were carried out in an MJ Research DNA Engine Dyad^® ^Peltier Thermal Cycler (Watertown, Massachusetts) using an initial denaturation at 95°C for 3 min, followed by 30 cycles of 96°C for 25 s, 50°C for 20 s, 60°C for 5 min and with a final elongation at 72°C for 7 min. Excess of dye terminators were removed using CleanSeq magnetic bead sequencing reaction clean up kit from Agencourt Biosciences (Beverly, MA). Sequences were resolved on an ABI 3730xl capillary-based automated DNA sequencer (Applied Biosystems) with 50 cm POP-7 polymer capillaries at the Biotechnology Center of the University of Wisconsin-Madison. When a faint second band appeared or when the sequence was unreadable, the PCR product was cloned and five positive colonies were sequenced. Subsequently we learned that 18 of these 19 COSII are single copy on tomato, 12 in potato and 11 in both potato and tomato (see Additional file 3).

### Sequence editing and alignment

Sequences were edited with Staden package version-2003.0-beta [46] and aligned using ClustalX version 1.81 [47] at default parameters, except for the "percentage of delay divergence sequences" which was set to 15% after tests of various parameters. Further manual alignments were done in MacClade 4.06 PPC [48] minimizing the number of gaps and preferring transitions to transversions. Indels were scored by the simple gap scoring method [49] using SeqState 1.40 [50]. DNA sequences were deposited in GenBank (see Additional file 4).

### Phylogenetic analyses

All analyses were conducted on three data sets: 1) a "potato data set" of 12 COSII across 11 species, nine of sect. *Petota *and two of sect. *Etuberosum*, 2) a "tomato data set" of 18 COSII across 19 species that included all species except members of sect. *Petota *and sect. *Etuberosum*, 3) the "complete data set" of 11 COSII across all 30 species. Phylogenetic analyses based on maximum parsimony were performed using PAUP* 4.0b10 [51]. The most parsimonious trees were found by heuristic searches under Fitch criteria and with equal weight for all characters. A heuristic search was performed using TBR branch swapping on 100,000 random taxon addition sequences. A rooted strict-consensus tree was obtained using *S. etuberosum *and *S. palustre *as outgroups for the potato data set, and *Datura inoxia *and *S. dulcamara *for the other two data sets. Bootstrap support [52] was estimated with 10,000 bootstrap replicates performing a TBR branch swapping on 100,000 random taxon addition sequences.

Maximum likelihood analysis was used to assess 56 models evaluated in Modeltest ver. 3.07 [53] using the Akaike Information Criterion (AIC) and hierarchical likelihood ratio test (hLRT) at α = 0.01. ML phylogeny was estimated using RAxML 7.0.3 [54] that allows each partition (each COSII) to have its own model and parameters. To evaluate the stability of clades on the optimal tree, a bootstrap analysis was performed with 100 bootstrap replicates.

Bayesian Markov chain Monte Carlo (MCMC) phylogenetic analysis [55] also was performed using MrBayes version 3.1.2 [56,57]. Model parameters for DNA data were chosen according to the criteria described above, they were estimated separately for DNA and gaps; for gaps the restriction site (binary) model was used (lset coding = variable) which accounts for the ascertainment bias produced by characters that are constant (either state 0 or 1) in all taxa and are not observed. Tree searching using MrBayes was performed by four runs of four linked chains ("temp" was empirically determined) for 1,100,000 or 2,200,000 generations with trees sampled every 100 generations. At the end of the analysis we checked that the average standard deviation of split frequencies was below 0.01 to ensure that convergence occurred properly. A conservative burn-in period was determined, and only post-burn-in trees were saved. The three sets of post-burn-in trees were then combined to form a majority rule consensus tree, and this pool was taken as the best representation of the posterior distribution of tree topology and model parameters [56]. The proportion of searches in which any given node is found during the post burn-in portion of the chain constitutes the Bayesian posterior probability (PP) for that node.

The ILD test [34] was used to explore the difference in phylogenetic signal between and among data partitions, and in the potato data set was used to implement the prior agreement approach, as implemented through the partition homogeneity test in PAUP* 4.0b10 [51]. Multiple ILD tests comparing all COSII sequenced in each data set were performed and the sequential Bonferroni correction was used to control for multiple comparisons [34,58]. First, we performed one ILD test to see if all COSII sequenced in each data set can be combined together. Given that they did not pass the test, we proceeded to make all the possible pairwise comparisons within each data set to identify the COSII that were congruent. A total of 66 pairwise comparisons were made for the potato data set, 153 for the tomato data set and 55 for the potato and tomato data set. The Templeton test [59] as implemented in PAUP* 4.0b10 [51] was used to explore alternative topologies in a parsimony framework.

To assess the total evidence approach we performed a single analysis with the concatenated data set. For the prior agreement approach we were able to identify groups of COSII that gave congruent results only with the potato data set. For the consensus approach, Bayesian concordance analysis (BCA) [36] was performed in the potato and tomato data sets separately, to identify the best estimate of the phylogeny of each COSII and to create a consensus of these separate point estimates. Three different prior levels of discordance: α = 1, 10, and infinite were used. For instance, an α value of zero corresponds to the total evidence approach that insists there is a single tree for all genes, and an infinite α value corresponds to an assumption of independence between gene trees. With the potato data set four independent runs with four linked chains were performed for all three α values; in each run with 1,100,000 generations, 100,000 of which were discarded as the burn-in period. For heating chains, the option used was m = 50. Runs showed good mixing and converged to the same results since the standard deviation of concordance factors was < 0.001 in all cases.

For tomato concordance analysis, it was impossible to perform a single concordance analysis with all of the species because the trees sampled during the Bayesian analysis of four COSII (COS13b, COS1b, COS4 and COS8) were all distinct, and it was necessary to prune some species to reduce the number of trees sampled in the Bayesian analysis to assure convergence to stationary distribution within an acceptable error. For this reason, we performed three independent analyses: 1) to determine the placement of the sections that are sister to tomatoes, a concordance analysis was performed with sequence of 18 COSII in 12 OTUs that included species or allelic variants of *Datura inoxia*, *S. dulcamara*, *S. lycopersicoides*, *S. sitiens*, *S. juglandifolium*, *S. ochranthum*, *S. pennellii*, *S. corneliomulleri*, *S. huaylasense*-1, *S. huaylasense*-2, *S. arcanum *and *S. lycopersicum*. For all three α values, four independent runs with four linked chains were performed. In each run of 22 million generations, 10% of the generations were discarded as the burn-in period; and for heating chains we used the option m = 50. Runs showed good mixing and converged to the same results since the standard deviation of concordance factors was < 0.005 in each case. 2) The second concordance analysis was conducted to determine if *S. habrochaites *and *S. pennellii *are the most divergent tomato species and to determine the placement of *S. chilense*, *S. corneliomulleri*, and *S. peruvianum*. We conducted a concordance analysis with all 18 COSII and with ten OTUs (*S. juglandifolium*, *S. pennellii*, *S. habrochaites*-1, *S. habrochaites*-2, *S. chilense*-1, *S. chilense*-2, *S. corneliomulleri*, *S. peruvianum*-1, *S. peruvianum*-2, and *S. lycopersicum*) using the same parameters that were used for the outgroups. Runs showed good mixing (SD of concordance factors was < 0.005 in each α) 3) The last concordance analysis with the tomato data set with all 18 COSII was conducted to determine the placement of *S. huaylasense*, and to determine relationships among *S. arcanum*, *S. chmielewskii*, and *S. neorickii*. Four independent runs of four linked chains were performed for each α with seven OTUs (*S. pennellii*, *S. huaylasense*-1, *S. huaylasense*-2, *S. neorickii*, *S. arcanum*, *S. chmielewskii *and *S. lycopersicum*).

## Authors' contributions

DS conceived and designed the research and obtained funding. FR designed the research, performed the DNA extractions, generated the DNA sequences, carried out the phylogenetic analyses and wrote the manuscript. FW and ST developed the COSII markers. CA performed the concordance analyses. FR and DS revised several versions of the manuscript. CA, FW and ST reviewed the last version of the manuscript. All the authors approved the final manuscript.

## Supplementary Material

Additional file 1 **Species examined; their superseries and series relationships within sect.***Petota*, endosperm balance number (EBN), genomes and plastid DNA clade relationships. Click here for file

Additional file 2 The 40 COSII oligonucleotide primers screened in this study. Click here for file

Additional file 3 The 19 COSII markers used in this study and their characteristics. Click here for file

Additional file 4 NCBI sequence database accession numbers. Click here for file
