# Supplementary material for: Do potatoes and tomatoes have a single evolutionary history, and what proportion of the genome supports this history?
Source: BMC Evol Biol. 2009 Aug 7;9:191. doi: 10.1186/1471-2148-9-191 (PMC3087518; doi:10.1186/1471-2148-9-191)
Supplement: Additional file 1 — Species examined; their superseries and series relationships within sect.Petota, endosperm balance number (EBN), genomes and plastid DNA clade relationships. [file 1471-2148-9-191-S1.doc]

**Additional file 1.** Species examined; their superseries and series relationships within sect. *Petota* according to Hawkes [4], Accession number or LA number from the C.M. Rick Tomato Genetics Resource Center or collection voucher, endosperm balance number (EBN), genomes [4,9] and plastid DNA clade relationships according to Spooner and Castillo [21].

| Sections or Series | Superseries (Hawkes, 1990) | Species | Accession | EBN | Genomes [9] | Genomes [4] | Plastid clade |
| --- | --- | --- | --- | --- | --- | --- | --- |
| Section *Petota* Dumort. |  |  |  |  |  |  |  |
| Ser*. Bulbocastana* (Rydb.) Hawkes | primitive Stellata Hawkes | *Solanum bulbocastanum* Dunal | 347757 | 1 | A^b^A^b^ | BB | 2 |
| Ser. *Megistacroloba* Cárdenas and Hawkes | primitive Rotata Hawkes | *S. raphanifolium* Cárdenas and Hawkes | 265862 | 2 | AA |  | 4 |
| Ser. *Pinnatisecta* (Rydb.) Hawkes | primitive Stellata | *S. stenophyllidium* Bitter | 255527 | 1 |  | BB | unknown |
|  | primitive Stellata | *S. trifidum* Correll | 255536 | 1 |  | BB | 1 |
| Ser. *Piurana* Hawkes | advanced Rotata | *S. albornozii*  Correll | 498206 | 2 |  |  | 3 |
| Ser. *Polyadenia* Correll | primitive Stellata | *S. polyadenium* Greenm. | 161728 | nd | A^po^A^po^ | BB | 1 |
| Ser. *Tuberosa* (Rydb.) Hawkes | primitive Rotata | *S. andreanum* Baker | 320345 | 2 | AA | A^1^A^1^ | 3 |
|  | primitive Rotata | *S. brevicaule* Bitter | 498115 | 2 | AA | A^1^A^1^ | 4 |
|  | advanced Rotata | *S. verrucosum* Schltdl. | 161173 | 2 | AA | A^1^A^1^ | 4 |
| Section *Etuberosum* |  | *S. etuberosum* Lindl. | 498311 | 1 | E^e^E^e^ |  |  |
| (Bukasov and Kameraz) A. Child |  | *S. palustre* Peopp. | 558233 | 1 | E^b^E^b^ |  |  |
| Section *Lycopersicon* (Miller) Wettstein |  | *S. arcanum* Peralta | LA 2185 |  |  |  |  |
|  |  | *S. cheesmaniae* (L. Riley) Fosberg | LA 1450 |  |  |  |  |
|  |  | *S. chilense* (Dunal) Reiche | LA 1963 |  |  |  |  |
|  |  | *S. chmielewskii* (C. M. Rick et al.) | LA 1306 |  |  |  |  |
|  |  | Spooner et al. |  |  |  |  |  |
|  |  | *S. corneliomulleri* J. F. Macbr. | LA 1283 |  |  |  |  |
|  |  | *S. galapagense* S. C. Darwin & Peralta | LA 0317 |  |  |  |  |
|  |  | *S. habrochaites* S. Knapp & D. M. Spooner | LA 1353 |  |  |  |  |
|  |  | *S. huaylasense* Peralta | LA 1982 |  |  |  |  |
|  |  | *S. lycopersicum* L. | LA 1673 |  |  |  |  |
|  |  | *S. neorickii* D. M. Spooner et al. | LA 1326 |  |  |  |  |
|  |  | *S. pennellii* Correll | LA 0716 |  |  |  |  |
|  |  | *S. peruvianum* L. | LA 2744 |  |  |  |  |
|  |  | *S. pimpinellifolium* L. | LA 1581 |  |  |  |  |
| Section  *Juglandifolia* (Rydberg) A. Child |  | *S. juglandifolium* Dunal | LA 2134 |  |  |  |  |
|  |  | *S. ochranthum* Dunal | LA 2682 |  |  |  |  |
| Section *Lycopersicoides* (A. Child) Peralta |  | *S. lycopersicoides* Dunal | LA 1964 |  |  |  |  |
|  |  | *S. sitiens* I. M. Johnst. | LA 2876 |  |  |  |  |
| Further outgroups |  | *S. dulcamara* L. | Spooner 2988 |  |  |  |  |
|  |  | *Datura inoxia* Mill. | Spooner 2989 |  |  |  |  |
