# Supplementary material for: Do potatoes and tomatoes have a single evolutionary history, and what proportion of the genome supports this history?
Source: BMC Evol Biol. 2009 Aug 7;9:191. doi: 10.1186/1471-2148-9-191 (PMC3087518; doi:10.1186/1471-2148-9-191)
Supplement: Additional file 2 — The 40 COSII oligonucleotide primers screened in this study. [file 1471-2148-9-191-S2.doc]

**Additional file 2.** The 40 COSII oligonucleotide primers screened in this study.

| COSII-marker | Primer Lab code | Forward Primer  (5 ' - 3') | Reverse Primer  (5 ' - 3') |
| --- | --- | --- | --- |
|
| U237757 | X1 | ATCGGCTGCTGATGTTTATGATCG | ACAACATCCCTCCATAGAGTTTCAAG |
| C2At1g30580 | X2 | TTCTGCCGAAGATTCATGCATGG | TCTCTCCACAGCAGCACTGAAAGG |
| C2At1g61620 | X3 | ATGCATTCTAGAATGCCTTTTGTC | TCCCTGGCTTTCTGCAGCATC |
| C2At4g38630 | X4 | TGGCTGGTAAAGGGGTTCGAG | AGCAAAAACAATAATCCTTTGTTG |
| U146140 | 1 | TTCCAGTGCGCTGTTTCACCTG | ATCTAACCAAATTCAGTGCATCAAG |
| C2At1g23740 | 2 | TGAAGTTGGAGTCCAATGTTGCAG | ACCGCCAGCACCTCCCAAAAC |
| C2At1g32130 | 3 | TCAACAAGAGTACACGGTTTGAAGAC | TTGCTCTAGCCCTGGCCCTAAC |
| C2At1g73180 | 4 | AGGACTTACAAAGTCAACCTGTTGCTC | ACCATTGAACATCATGAATAGGGC |
| C2At2g15890 | 5 | AGCCTATTTTGAACTCAAAGATCTTG | TTCTCTCGACTTTGGCAATCCATC |
| C2At2g20930 | 6 | ATCACATTGTCCATTGCTCCCTTG | TTCACTTTTGTATTAGTCAAATAACC |
| C2At2g22570 | 7 | ACTGAAGAGTGAGATTCCGGTGGAG | TCTGTTCCAGTGATACAATGAGGAGG |
| C2At2g36930 | 8 | TCACAGGAGCGTGAAGAAGCG | TCTTCGTCTTGAAATGCTCATCCC |
| C2At2g38020 | 9 | TGCAGCTTTGCTTTATGATGCC | AAAGGCTTGGCCGTAGCTTGC |
| U146437 | 10 | TGCACGAAGACACAGCTTGGC | AACCCAACCATGGCATTAACC |
| C2At5g14320 | 11 | TTCTCTTTCCCTTATCTGCAACAC | TCCTTCAATCATGTACTTAGAGACTTC |
| C2At5g58490 | 12 | TACTTGGTTTAGAAGGAGCTGAATC | ATACAATCAGCTGGCCAATTAGG |
| C2At1g16210 | 1B | AGGAAGGAGGCGGAGCAGGC | TCCTTCAGAGCACGAAGCTCAGC |
| C2At1g29990 | 2B | TTGGTGAAAACGAGCTTGTTCTC | TAAACATTGGCGTCTTCATTCAAC |
| C2At1g44575 | 3B | AGCTATTTGTTGGCCGTGTTGC | TGCCTCTGTCTCCCAAAGCTCC |
| C2At1g44760 | 4B | TTCTTCATCTGCTGCTCATCTTGC | AGAGGGTTTTTTCTGACCCAAGAC |
| C2At1g49670 | 5B | ACCCTGGAATATGCGAAAAGCTTC | TCTTGCCATAGGTGAGCATATTG |
| C2At1g76080 | 6B | TAGTATGGAGGAATTGGATGAAGC | TCTTCTCTGCTGTGGAGCTGCAC |
| C2At1g77470 | 7B | TGCCCTACAATCACGATGTACACG | AAACCACCCTCAGGGACATCAAG |
| C2At2g24270 | 8B | TGGAAATGAAAGGACCAAATACTG | AGGAGCAATCTTGGAGACGGC |
| C2At3g03100 | 9B | TGGTGCAACACTTGTTGGTGTGG | TGGAGCCAGCCATGCCATTC |
| C2At3g10920 | 10B | TGGCTTGGTGTGGACAAAGAGC | TGCAAGTAGTATGCGTGTTCCC |
| C2At3g16150 | 11B | AAGCAACTCCTTACTCGTTGCC | TGGAGATACCAGAAACGGCG |
| C2At3g24530 | 12B | ATATGGTTGGTGAATTTGTTGGTC | AGAATCCCCCCTTCAGCTTCTTG |
| C2At3g55800 | 13B | TTTGAAATCAAGCTCATTATTTGG | AGCTGTTCCTCCACAAGAAGCTG |
| C2At4g20150 | 14B | TTCGAAAACTTCCTTACATGCG | TCCCATACCCAACAAATGTTCC |
| C2At4g34700 | 15B | TGAAGCATCCTACAATAAGTGGCG | TCTGTTGAACTTGGAACCACCAGG |
| C2At5g27620 | 16B | ATCTACAATGGTCCGTGATGGAAC | TTCCTCTGCCTTGCAAGCTGC |
| C2At1g13380 | 1C | AGGTGCTTTCTTGTTTCTTCTTTC | AGAGCATATCACGATACTTGGTGTG |
| C2At1g14000 | 2C | TTCGCAAATACTCTGGCACGCTC | ATACGTCTTTAACAATTCAATCATGC |
| C2At1g20050 | 3C | ATGATCTAAAATTGCCTGGTTTTG | AATAGCCCTCAAGGACCATGTGG |
| U217233 | 4C | ACGCTAAGTTCGCCGGAAAGC | TCCTTGGAACGTGCAGCAGATTGTC |
| C2At1g50020 | 5C | TTGCTTACTCTTGGTGGAACATTC | TGTCTGTGATATCCTCTCTTCTTC |
| U221402 | 6C | AAGCCTCCTTGACAAATGCATATAG | AGATATAGCTACAGTGGCAGCTTCATC |
| C2At2g41170 | 7C | AGGAGGGCAATGGCAATAGAGACAG | ATTTCCATCACACGTTTCCAAGTG |
| C2At2g46220 | 8C | TCTGAATACCGGTTCTGCTATTCG | TGCCTTCCACAGGCTGCCATACAC |
