# Supplementary material for: Do potatoes and tomatoes have a single evolutionary history, and what proportion of the genome supports this history?
Source: BMC Evol Biol. 2009 Aug 7;9:191. doi: 10.1186/1471-2148-9-191 (PMC3087518; doi:10.1186/1471-2148-9-191)
Supplement: Additional file 3 — The 19 COSII markers used in this study and their characteristics. [file 1471-2148-9-191-S3.doc]

**Additional file 3.** The 19 COSII markers used in this study and their characteristics.

^1^ Database at Sol Genomics Network.

^2^ Range for the best annealing temperature.

^3^ F_2_ map position in the mapping population Tomato EXPEN 2000 ([http://www.sgn.cornell.edu](http://www.sgn.cornell.edu/)).

^4^ s denotes single copy, m multiple copy, ? unknown.

| **COSII marker** | **Primer Lab code** | **Results from agarose gel** | | | | | **T ann^2^ °C** | **Chrm^3^** | **Map position****^3^ cM** | **no. copies^4^** | |
| --- | --- | --- | --- | --- | --- | --- | --- | --- | --- | --- | --- |
|  |  | **no. bands** | **Exon^1^ bp** | **Total bp** | **Intron bp** | **Intron %** |  |  |  | **tom** | **pot** |
| **C2At1g30580** | **X2** | single | 77 | 1,000 | 923 | 92 | 55 | 2 | 0.00 | s | m |
| **C2At1g32130** | **3** | single | 189 | 480 | 291 | 61 | 56 | unknown |  | s | s |
| **C2At1g73180** | **4** | single | 169 | 300 | 131 | 44 | 54 | unknown |  | s | ? |
| **C2At2g15890** | **5** | single | 229 | 850 | 621 | 73 | 52-55 | 1 | 150.00 | s | s |
| **C2At2g36930** | **8** | single | 227 | 300 | 73 | 24 | 54 | 9 | 15.10 | s | s |
| **C2At2g38020** | **9** | single | 187 | 500 | 313 | 63 | 55-56 | 7 | 49.00 | s | s |
| **C2At5g14320** | **11** | single | 139 | 700 | 561 | 80 | 50-51 | 5 | 10.00 | s | s |
| **C2At1g16210** | **1B** | single | 269 | 400 | 131 | 33 | 55 | 1 | 165.00 | s | m |
| **C2At1g77470** | **7B** | single | 118 | 1,500-2,000 | 1,382-1,882 | 92-94 | 55 | 6 | 39.10 | s | s |
| **C2At2g24270** | **8B** | single | 112 | 700-1,000 | 588-888 | 84-89 | 55 | 7 | 0.30 | s | m |
| **C2At3g03100** | **9B** | single | 153 | 1,000-1,200 | 847-1,047 | 85-87 | 55-56 | 3 | 72.70 | s | s |
| **C2At3g10920** | **10B** | single | 135 | 700 | 565 | 81 | 55-56 | 6 | 13.00 | s | s |
| **C2At3g16150** | **11B** | single | 221 | 400 | 179 | 45 | 55-56 | 4 | 97.50 | s | m |
| **C2At3g55800** | **13B** | single | 241 | 300 | 59 | 20 | 55-56 | 5 | 90.00 | s | m |
| **C2At4g34700** | **15B** | single | 80 | 1,000 | 920 | 92 | 55-56 | 1 | 160.00 | s | m |
| **C2At1g13380** | **1C** | single | 212 | 750 | 538 | 72 | 54-55 | 5 | 38.70 | s | s |
| **C2At1g14000** | **2C** | single | 145 | 800 | 655 | 82 | 55 | 5 | 59.50 | m | s |
| **C2At1g20050** | **3C** | single | 201 | 900-1,000 | 699-799 | 78-80 | 55 | 6 | 101.00 | s | s |
| **C2At1g50020** | **5C** | single | 189 | 1,100 | 911 | 83 | 55 | 1 | 146.10 | s | s |
