# Supplementary material for: Do potatoes and tomatoes have a single evolutionary history, and what proportion of the genome supports this history?
Source: BMC Evol Biol. 2009 Aug 7;9:191. doi: 10.1186/1471-2148-9-191 (PMC3087518; doi:10.1186/1471-2148-9-191)
Supplement: Additional file 4 — NCBI sequence database accession numbers. [file 1471-2148-9-191-S4.doc]

**Additional file 4.** NCBI sequence database accession numbers.

| | **COSII marker** | **Primer Lab code** | | **Potato dataset** | | | **Tomato dataset** | | | | --- | --- | --- | --- | --- | --- | --- | --- | --- | | | **C2At1g30580** | **X2** |  | |  | FJ599002-FJ599021 | | |  | | **C2At1g32130** | **3** | FJ599275-FJ599285 | | | FJ598922-FJ598940 | | |  | | **C2At1g73180** | **4** |  | |  | FJ599043-FJ599061 | | |  | | **C2At2g15890** | **5** | FJ599308-FJ599318 | | | FJ599083-FJ599101 | | |  | | **C2At2g36930** | **8** | FJ599319-FJ599329 | | | FJ599124-FJ599142 | | |  | | **C2At2g38020** | **9** | FJ599330-FJ599340 | | | FJ599143-FJ599161 | | |  | | **C2At5g14320** | **11** | FJ599363-FJ599373 | | | FJ599223-FJ599241 | | |  | | **C2At1g16210** | **1B** |  | |  | FJ598963-FJ598982 | | |  | | **C2At1g77470** | **7B** | FJ599297-FJ599307 | | | FJ599062-FJ599082 | | |  | | **C2At2g24270** | **8B** |  | |  | FJ599102-FJ599123 | | |  | | **C2At3g03100** | **9B** | FJ599341-FJ599351 | | | FJ598903-FJ598921 | | |  | | **C2At3g10920** | **10B** | FJ599352-FJ599362 | | | FJ599162-FJ599181 | | |  | | **C2At3g16150** | **11B** |  | |  | FJ599182-FJ599201 | | |  | | **C2At3g55800** | **13B** |  | |  | FJ599202-FJ599222 | | |  | | **C2At4g34700** | **15B** |  | |  | FJ598941-FJ598962 | | |  | | **C2At1g13380** | **1C** | FJ599242-FJ599252 | | | FJ598884-FJ598902 | | |  | | **C2At1g14000** | **2C** | FJ599253- FJ599263 | | |  | |  |  | | **C2At1g20050** | **3C** | FJ599264-FJ599274 | | | FJ598983-FJ599001 | | |  | | **C2At1g50020** | **5C** | FJ599286-FJ599296 | | | FJ599022-FJ599042 | | |  | |
| --- | --- | --- | --- | --- | --- | --- | --- | --- | --- | --- | --- | --- | --- | --- | --- | --- | --- | --- | --- | --- | --- | --- | --- | --- | --- | --- | --- | --- | --- | --- | --- | --- | --- | --- | --- | --- | --- | --- | --- | --- | --- | --- | --- | --- | --- | --- | --- | --- | --- | --- | --- | --- | --- | --- | --- | --- | --- | --- | --- | --- | --- | --- | --- | --- | --- | --- | --- | --- | --- | --- | --- | --- | --- | --- | --- | --- | --- | --- | --- | --- | --- | --- | --- | --- | --- | --- | --- | --- | --- | --- | --- | --- | --- | --- | --- | --- | --- | --- | --- | --- | --- | --- | --- | --- | --- | --- | --- | --- | --- | --- | --- | --- | --- | --- | --- | --- | --- | --- | --- | --- | --- | --- | --- | --- | --- | --- | --- | --- | --- | --- | --- | --- | --- | --- | --- | --- | --- | --- | --- | --- | --- | --- | --- | --- | --- | --- | --- | --- | --- | --- | --- | --- | --- | --- | --- | --- | --- | --- | --- | --- | --- | --- | --- | --- | --- | --- | --- | --- | --- | --- | --- | --- | --- | --- | --- | --- | --- | --- | --- | --- |
